# Neurofilament light chain but not glial fibrillary acidic protein serum levels are elevated in Wolfram syndrome

**DOI:** 10.3389/fnins.2026.1805916

**Published:** 2026-03-18

**Authors:** Matthew J. Jansen, Heather M. Lugar, Cris M. Brown, Abby F. Tang, Liam J. Oiknine, Ling Chen, Jonathan M. Koller, Brian A. Gordon, Bess A. Marshall, Fumihiko Urano, Tamara Hershey

**Affiliations:** 1 Department of Psychiatry, Washington University School of Medicine, St. Louis, MO, United States; 2 Department of Medicine, Washington University School of Medicine, St. Louis, MO, United States; 3 Institute for Informatics, Data Science, and Biostatistics, Washington University School of Medicine, St. Louis, MO, United States; 4 Mallinckrodt Institute of Radiology, Washington University School of Medicine, St. Louis, MO, United States; 5 Department of Psychological and Brain Sciences, Washington University in St. Louis, St. Louis, MO, United States; 6 Department of Pediatrics, Washington University School of Medicine, St. Louis, MO, United States; 7 Department of Cell Biology, Washington University School of Medicine, St. Louis, MO, United States; 8 Department of Pathology and Immunology, Washington University School of Medicine, St. Louis, MO, United States

**Keywords:** blood-based biomarkers, glial fribrillary acidic protein (GFAP), neurodegeneration, neurofilament light chain (NFL), neuroimaging, Simoa assay, WFS1, Wolfram syndrome

## Abstract

**Background:**

Wolfram syndrome is a rare genetic disorder caused by pathogenic variants in the *WFS1* gene. Progressive neurodegeneration, a key feature of the disease, is an important target of current and future clinical trials. Serum neurofilament light chain (NfL) and glial fibrillary acidic protein (GFAP) are promising blood-based biomarkers of neuroaxonal damage and reactive astrogliosis, respectively, that may be useful alternative or adjunctive outcome measures to current measures of disease progression.

**Objective:**

To determine if serum NfL and/or GFAP levels are elevated in Wolfram syndrome compared to controls and whether they can serve as monitoring biomarkers.

**Methods:**

Serum NfL and GFAP levels were log_10_ transformed and compared between individuals with Wolfram syndrome (*n* = 45) and multiple control groups, including their parents (*n* = 55), unaffected siblings (*n* = 12), and unrelated individuals with (*n* = 47) and without (*n* = 29) newly diagnosed Type 1 diabetes. Within the Wolfram group, serum levels were related to clinical measures and regional brain volumes and assessed longitudinally.

**Results:**

NfL levels were higher in the Wolfram group relative to all control groups (*p* < 0.001, $\eta_{p}^{2}$ = 0.51) after adjusting for age and sex, whereas GFAP levels were not different between any of the groups. Within the Wolfram group, neither NfL nor GFAP levels changed over time, and NfL levels did not correlate reliably with any measures of clinical disease severity or neurodegeneration (*p* > 0.05 after excluding outliers).

**Conclusion:**

Serum NfL elevation in Wolfram syndrome may reflect the ongoing, relatively slow neurodegeneration occurring in this disorder. However, without any correspondence between serum levels and currently used clinical and neuroimaging metrics, it has limited utility as a monitoring biomarker of disease progression in this patient population. Future studies may be warranted to determine if NfL could be a treatment-response marker in Wolfram syndrome clinical trials.

## Introduction

Wolfram syndrome is an extremely rare genetic disorder with an estimated global prevalence ranging from 1 in 100,000 (Fraser and Gunn, 1977) to 1 in 770,000 (Barrett et al., 1995). It is primarily characterized by childhood-onset insulin-dependent diabetes mellitus, optic nerve atrophy, sensorineural hearing loss, central diabetes insipidus (arginine vasopressin deficiency), and neurodegeneration (Barrett et al., 1995; Urano, 2016) with neurologic symptoms typically arising in early childhood and progressing over time, leading to impairment and a shortened lifespan (Barrett et al., 1995; Urano, 2016). The classical form of Wolfram syndrome is caused by biallelic pathogenic variants in the *WFS1* gene (Inoue et al., 1998), which encodes the transmembrane endoplasmic reticulum (ER) glycoprotein wolframin. Wolframin is thought to play a role in intracellular calcium homeostasis; deficient wolframin levels may compromise ER function resulting in mitochondrial dysfunction and ER stress-mediated apoptosis (Riggs et al., 2005; Yamada et al., 2006; Fonseca et al., 2010).

Findings from our long-standing natural history study of Wolfram syndrome in children, adolescents, and young adults have revealed early-onset deficits in vision, balance, smell identification, and hearing (Pickett et al., 2012; Karzon et al., 2018; Alfaro et al., 2020; O’Bryhim et al., 2022) and reduced regional volumes in the pons, cerebellar white matter, thalamus, optic nerve and total intracranial volume (ICV) (Hershey et al., 2012; Hoekel et al., 2014; Lugar et al., 2016, 2019; Samara et al., 2020). Longitudinal follow-up of this cohort has shown ongoing neurodegeneration, represented by continued reduction of regional brain volumes and key symptoms over years (Hoekel et al., 2018; Lugar et al., 2019). Clinical trials targeted at slowing or stopping neurodegeneration in Wolfram syndrome would benefit from more accessible and more easily measured biomarkers of neurodegeneration than costly and complicated magnetic resonance imaging (MRI) scans. Fluid biomarkers of neuroaxonal damage and reactive astrogliosis, such as neurofilament light chain (NfL) and glial fibrillary acidic proteins (GFAP), respectively, have been shown to be useful in other more common neurodegenerative diseases (Abdelhak et al., 2022; Heimfarth et al., 2022; Khalil et al., 2024), but have not been fully examined in Wolfram syndrome for these purposes.

NfL proteins are key components of the axonal cytoskeleton that help facilitate axonal branching and maintain neuronal structural integrity (Yuan et al., 2012, 2015, 2017; Yuan and Nixon, 2016; Gaetani et al., 2019) and are released into cerebrospinal fluid (CSF) and transit to blood in normal aging and upon neuroaxonal damage in various neurological conditions, such as stroke, trauma, and dementia (Khalil et al., 2024). The recent introduction of ultrasensitive single-molecule array (Simoa) (Wilson et al., 2016) assays has made it possible to reliably measure NfL in the blood in both healthy and diseased states, bypassing the need for invasive lumbar puncture to obtain CSF NfL levels (Khalil et al., 2024). Elevated NfL levels in the blood or CSF indicate neuroaxonal damage and correlate with clinical severity and MRI findings in various neurodegenerative diseases (Khalil et al., 2024). Although NfL levels are nonspecific and independent of specific disease etiology, they are sensitive to neurodegeneration and may serve as a marker of onset and progression across many neurological disorders, such as multiple sclerosis (MS) in adults and children, Alzheimer’s disease (AD), and spinocerebellar ataxia (Disanto et al., 2017; Reinert et al., 2020; Coarelli et al., 2021; Fuloria et al., 2026). A previous pilot analysis from our group showed that plasma NfL levels were significantly elevated in children and young adults with Wolfram syndrome compared to family members, primarily parents, without Wolfram syndrome (Eisenstein et al., 2022) and correlated with greater disease severity and faster annual rate of volume loss in the thalamus over 2 years. However, this analysis could not control for the effects of insulin dependent diabetes (Sampedro et al., 2020) or kidney dysfunction (Van Der Plas et al., 2022) and the longitudinal follow-up was limited to a two-year span.

GFAP is the primary intermediate filament of astrocytes, glial cells that help regulate synaptic transmission and provide structural and metabolic support to neurons (Abdelhak et al., 2022). Similar to NfL, GFAP is released into the CSF and transits to blood in normal aging (Verberk et al., 2021; Abdelhak et al., 2022) and at higher levels upon CNS injury or disease (Petzold, 2015; Abdelhak et al., 2022; Heimfarth et al., 2022). It can be detected in the blood by ultra-sensitive immunoassay platforms (Abdelhak et al., 2022) and has demonstrated wide use as a potential biofluid biomarker for reactive astrogliosis in neurological diseases, including traumatic brain injury (TBI) (Bazarian et al., 2025), MS (Chitnis et al., 2025) and AD (Kim et al., 2023). Plasma GFAP has also demonstrated utility as a prognostic and monitoring biomarker of neuroimaging-based changes in AD/mild cognitive impairment, Parkinson’s disease, frontotemporal dementia, and cerebrovascular disease (Sanchez et al., 2025). Neurological disorders that share phenotypic similarities with Wolfram syndrome, such as neuromyelitis optica spectrum disorder (NMOSD), also display elevated serum GFAP (Watanabe et al., 2019; Schindler et al., 2021). Thus, it is plausible that GFAP levels will be elevated in Wolfram syndrome relative to controls.

The primary aim of this study was to determine if serum NfL and/or GFAP levels are higher in Wolfram syndrome compared to controls both cross-sectionally and longitudinally and after controlling for relevant confounds. Determining if NfL or GFAP levels correlate with or predict changes in neurologic symptoms and regional brain volumes over time in Wolfram syndrome would support the use of these blood-based measures for disease and treatment monitoring in clinical trials.

## Methods

### Participants

All studies were approved by the Human Research Protection Office at Washington University in St. Louis, and methods were conducted in accordance with relevant guidelines and regulations. Children under age 18 gave informed assent, and parents/guardians gave informed, written consent. Participants 18 or older gave informed, written consent.

#### Wolfram syndrome group

Individuals diagnosed with Wolfram syndrome (WFS) were recruited into the Washington University Wolfram Syndrome Research Clinic through self or physician referral or from the Washington University Wolfram Syndrome Registry. Inclusion criteria were genetically confirmed biallelic *WFS1* disease-causing variants, under the age of 30 at enrollment and aware of their diagnosis, and able to travel to St. Louis for annual research clinic visits. Enrolled participants underwent annual evaluations between 2010 and 2023. At each visit, fasting blood draws were taken and those without contraindications underwent magnetic resonance imaging (MRI). Some of the data acquired during these research clinics have been previously published (e.g., Hershey et al., 2012; Nguyen et al., 2012; Pickett et al., 2012; Karzon and Hullar, 2013; Karzon et al., 2018; Marshall et al., 2013; Hoekel et al., 2014, 2018; Bischoff et al., 2015; Lugar et al., 2016, 2019; Alfaro et al., 2020, 2023; Samara et al., 2020; Eisenstein et al., 2022; O’Bryhim et al., 2022). Data for the current analyses were acquired beginning as early as 2012, over a span of 11 years, and represent up to 6 different visits per participant.

#### Comparison groups

1. Biological parents and unaffected siblings of Wolfram participants. Biological parents and unaffected (no Wolfram syndrome symptoms) siblings of participants in the Wolfram group who accompanied their family member(s) between 2014 and 2023 were invited to participate. Some of the siblings had already undergone genetic testing and were determined to be carriers or have no *WFS1* mutations. Both parents and unaffected siblings had fasting blood draws taken.
2. Unrelated controls, with and without newly diagnosed Type 1 diabetes (T1D). In a separate study, participants with newly diagnosed T1D and controls were asked to provide a fasting plasma sample and perform other assessments (not reported here—Clinicaltrials.gov NCT03335878). The T1D group was assessed approximately 3 months and 21 months after their diagnosis. Participants were excluded if they had a diagnosed psychiatric disorder, significant neurological history not due to diabetes, known premature birth with complications, psychoactive medications, or physical limitations that would interfere with testing. No participants had known retinopathy, nephropathy, or neuropathy at the time of testing. Fasting blood draws were taken at visits between 2017 and 2021 and stored for later analysis.

### Measures

#### Serum NfL and GFAP levels

Blood was drawn from all participants at the time of each research study visit, after they had fasted, and then spun down and frozen at −80° C. Samples were sent to Quanterix for processing at the same time. Each sample was assayed for both NfL and GFAP using the Simoa® Neurology 2-Plex B (N2PB) Kit on the HD-X Analyzer platform according to manufacturer instructions. Two replicates per sample were averaged, and a coefficient of variance (CV) was calculated. Samples with a CV > 25% were excluded from analyses. Number of years that had passed since the blood sample collection was calculated.

#### Clinical measures

Key measures of clinical disease severity and neurodegeneration were chosen for analysis in relation to serum biomarkers that were significantly different across groups. In the Wolfram syndrome group, we used the Wolfram Unified Rating Scale (WURS) physical subscale (Nguyen et al., 2012; Bischoff et al., 2015), best-corrected visual acuity using a Snellen Optotype (logMAR score, both eyes open) (Hoekel et al., 2014, 2018; O’Bryhim et al., 2022), and total score on the University of Pennsylvania Smell Identification Test (UPSIT) (Doty et al., 1984; Alfaro et al., 2023). To assess kidney function in Wolfram syndrome participants, diagnosis with diabetes insipidus at their first timepoint and creatinine levels from fasting blood samples at available timepoints were used. For participants with diabetes (Wolfram and T1D group), hemoglobin A1c (HbA1c) values from fasting blood samples at their first timepoint were used.

#### Regional brain volumes

MRI scans were acquired in participants with Wolfram syndrome without contraindications for scanning. Scans performed at the same timepoints as serum samples were analyzed for key regional brain volumes known to decrease over time in Wolfram syndrome compared to controls, namely the thalamus, pons, brainstem, cerebellar gray and cerebellar white matter (Hershey et al., 2012; Lugar et al., 2016, 2019; Samara et al., 2020). All timepoints analyzed were acquired at the same site. For timepoints between 2012 and 2017, a Siemens 3T Tim Trio scanner was used to acquire the T1-weighted Magnetization-Prepared Rapid Gradient-Echo (MPRAGE) sequence in sagittal orientation with a repetition time (TR) = 2,400 ms, echo time (TE) = 3.16 ms, inversion time (TI) = 1,000 ms, voxel resolution = 1 mm^3^, and duration = 8:09 min. For timepoints between 2019 and 2023, a Siemens 3T Prisma scanner was used to acquire a multi-echo MPRAGE sequence in sagittal orientation with a TR = 2,500 ms, TE1 = 1.81 ms, TE2 = 3.60 ms, TE3 = 5.39 ms, TE4 = 7.18 ms, TI = 1,000 ms, voxel resolution = 0.8 mm^3^, duration = 7:07–8:22 min using volumetric navigators (vNav) to correct for motion (Tisdall et al., 2016). A longitudinal multi-scanner harmonization method called ComBat was used to correct for systematic, technical variability in scanner effects, such as scanner, software version, sequence and head coil type (Beer et al., 2020). Some Wolfram participants were scanned only on the Trio, some only on the Prisma, and some had timepoints spanning both. The semi-automatic segmentation program FreeSurfer (v8.0.0) was used to extract regional gray and white matter volumes of interest at each timepoint using the longitudinal processing stream (Fischl et al., 2002; Reuter et al., 2012). Regional volumes were averaged across left and right hemispheres when appropriate and corrected for estimated total intracranial volume (eTIV) (Buckner et al., 2004).

### Statistics

Raw serum NfL and GFAP levels were log_10_-transformed to normalize distributions (e.g., Schindler et al., 2021), and outliers greater than 3 standard deviations (SD) from the mean were excluded. Using an analysis of covariance (ANCOVA), NfL and GFAP levels were then compared cross-sectionally between the Wolfram group and the control groups using each participant’s first available timepoint, adjusting for age and sex. Demographic and clinical variables available across all groups, as well as diabetes variables between Wolfram and T1D, were also compared. Analyses with a significant group effect at *p* < 0.05 were followed by pairwise comparisons.

Longitudinal serum NfL and GFAP levels within the Wolfram group were assessed using a random coefficients mixed effects model in which serum level was modeled as a function of time. A main effect of time at *p* < 0.05, after adjusting for age and sex, was considered significant.

Average NfL and GFAP levels were also calculated for each Wolfram participant across all available timepoints. For serum, clinical, and regional brain variables, rate of change over time was calculated by fitting a first-degree polynomial to each participant’s longitudinal data to calculate slope with respect to age in years.

For serum measures (NfL and/or GFAP) that were elevated in the Wolfram group, the following correlations were explored: (1) Serum level(s) with measures of clinical and regional brain variables both at the first timepoint, and (2) Average serum level(s) with rate of change in measures of clinical and brain variables. In addition, potential effects of kidney function in Wolfram participants were considered using available creatinine levels and a diabetes insipidus diagnosis at their first timepoint.

## Results

### Participants

Descriptive statistics for the control and Wolfram groups are shown in Table 1. There was a difference in mean age between groups, *F* (4,183) = 320.81, *p* < 0.001, in which Wolfram parents were older than all other groups (*p* < 0.001), and the Wolfram group was older than the healthy control group (*p* = 0.013), but the other groups were not different from one another (*p* ≥ 0.176 for all other group comparisons). Groups also differed significantly by race and ethnicity, χ ^2^ (12) = 44.70, *p* < 0.001 and χ ^2^ (8) = 47.43, *p* < 0.001, respectively, but not sex χ ^2^ (4) = 7.00, *p* = 0.136. There was also a difference between groups in how much time had passed since the blood samples were collected, *F* (4,183) = 14.60, *p* < 0.001. Wolfram samples were collected longer ago than the other groups on average (*p* ≤ 0.002 for all of these comparisons), except for their siblings (*p* = 0.097); and the Wolfram group, parents, and siblings were all collected longer ago than the healthy and T1D control groups (*p* ≤ 0.023 for all of these comparisons). Both diabetes-related variables examined were higher within the Wolfram group when compared to the T1D group (HbA1c: *t* (1, 90) = 2.92, *p* = 0.004; diabetes duration: *t* (1, 86) = 10.73, *p* < 0.001).

**Table 1:** Demographics, clinical characteristics, and serum NfL and GFAP levels by group.

Measures	Wolfram	Wolfram Parent	Wolfram Sibling	Healthy Control	T1D	Group (*p*)
*N*	45	55	12	29	47	–
Male/Female	19/26	21/34	7/5	17/12	27/19	0.136
Race (W/B/MR/UNK)	45/0/0/0	45/0/0/10	11/0/0/1	21/7/0/1	39/3/2/2	**<0.001**
Ethnicity (NH/H/UNK)	29/16/0	30/15/10	9/2/1	27/2/0	45/1/0	**<0.001**
Mean ± SD						
Time since sample (years)	8.8 ± 2.9	7.31 ± 3.0	7.5 ± 4.1	5.1 ± 0.4	5.7 ± 0.8	**<0.001**
Diabetes duration (years)	9.2 ± 5.6	–	–	–	0.5 ± 0.5	**<0.001**
HbA1c (%)	7.7 ± 1.8	–	–	–	6.9 ± 1.2	**0.004**
Median (IQR)						
Age (years)	13.8 (9.5–18.2)	46.1 (40.8–51.4)	11.9 (8.6–15.3)	14.0 (11.8–16.2)	11.2 (8.5–13.9)	**<0.001**
NfL Raw (pg/mL)	11.5 (7.9–15.2)	6.9 (4.2–9.5)	4.1 (3.2–5.0)	3.5 (2.6–4.4)	4.0 (3.0–5.0)	<0.001*
NfL log10	1.1 (0.9–1.2)	0.8 (0.7–1.0)	0.6 (0.5–0.7)	0.5 (0.4–0.7)	0.6 (0.5–0.7)	<0.001*
GFAP Raw (pg/mL)	117.9 (77.4–158.3)	77.4 (55.1–99.7)	85.4 (62.2–108.5)	103.3 (74.0–132.5)	124.2 (72.8–175.5)	0.080*
GFAP log10	2.1 (1.9–2.2)	1.9 (1.8–2.0)	1.9 (1.8–2.0)	2.0 (1.9–2.1)	2.1 (1.9–2.3)	0.235*

NfL, neurofilament light chain; GFAP, glial fibrillary acidic protein; W, White; B, Black; MR, Mixed Race; UNK, Unknown; NH, Non-Hispanic; H, Hispanic; IQR, interquartile range; – indicates not applicable. * indicates age and sex used as covariates; Bold *p*-values indicate statistical significance at *α* = 0.05.

### Quality control

There were no statistical outliers within the Wolfram group’s longitudinal serum NfL and GFAP levels. One GFAP sample from the T1D group had a CV of 31% and one GFAP sample from a Wolfram participant’s third timepoint had a CV of 30%. Both were excluded from further analyses. The average, cross-sectional CV across 184 replicate samples was 5.8% for serum NfL levels and 6.7% for serum GFAP levels. The average longitudinal CV across 153 replicate samples was 3.64% for serum NfL levels and 6.75% across 152 replicate samples for serum GFAP. Three replicates from the cross-sectional sample were lost during processing (one from the parent group and two from the T1D group), while one longitudinal sample from the Wolfram group was lost. However, due to the high reliability and consistency of the data, as indicated by the low CVs, these four single values were retained in analyses.

### Group differences in NfL and GFAP

**Figure 1:**
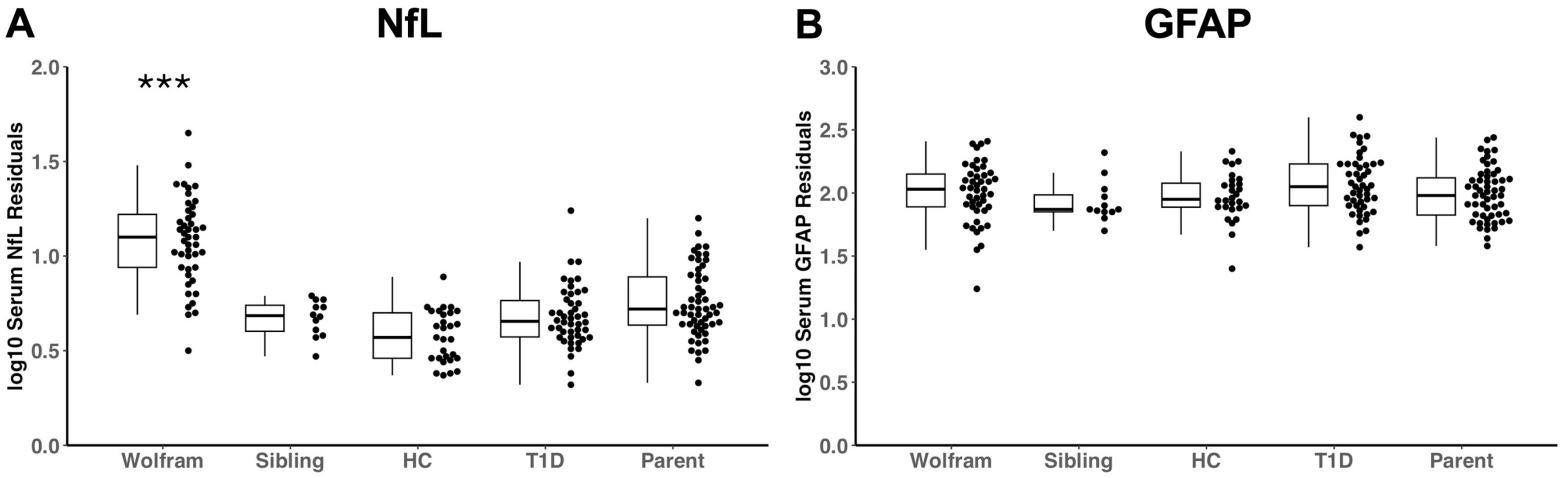
Log_10_ serum NfL levels **(A)** but not GFAP levels **(B)** were significantly higher in the Wolfram group at time 1 relative to all control groups adjusting for age and sex. Median and interquartile ranges (IQR) of residuals from a linear regression model adjusting for age and sex are shown. NfL, neurofilament light chain; GFAP, glial fibrillary acidic protein; HC, healthy control; T1D, type 1 diabetes. ***p < 0.001 relative to all control groups.

There was a main effect of group on log_10_ serum NfL at time 1 [log_10_: *F* (4,180) = 46.09, *p* < 0.001, $\eta_{p}^{2}$ = 0.51] when covarying for age and sex, as shown in Table 1 and Figure 1. *Post-hoc* comparisons revealed that NfL levels were significantly higher in the Wolfram group compared to each control group (*p* < 0.001 for all comparisons) with marginal means ranging from 57–85% higher in the Wolfram group. Log_10_ NfL levels were also higher in the T1D group relative to the healthy control group (*p* = 0.015). Even when adding race, ethnicity, and time since the sample was collected as additional covariates, the Wolfram group still had higher NfL levels than all other groups [log_10_: *F* (4, 177) = 32.09, *p* < 0.001, $\eta_{p}^{2}$ = 0.42, with means 21–98% higher in Wolfram].

To determine if diabetes severity influenced NfL levels, we compared the Wolfram and T1D group while covarying age, sex, race, ethnicity, time since sample was collected, plus HbA1c and diabetes duration. Higher NfL levels in the Wolfram group remained (log_10_: *F* (1, 78) = 28.97, *p* < 0.001, $\eta_{p}^{2}$ = 0.27, with the mean 72% higher in Wolfram).

To determine if kidney function influenced NfL levels, we compared NfL levels in Wolfram participants with and without a diabetes insipidus diagnosis, while controlling for age and sex; no difference was found [log_10_: *F* (1, 41) = 1.21, *p* = 0.279]. Creatinine levels were available for 38 out of the total 45 Wolfram participants for at least one timepoint. Participants’ levels were within normal limits for their age except for three individuals whose levels were above the reference range.

No main effect of group on log_10_ serum GFAP was found [log_10_: *F* (4,180) = 1.40, *p* = 0.235, $\eta_{p}^{2}$ = 0.03] when covarying for age and sex, or when covarying for age, sex, race, ethnicity, and time since sample [log_10_: *F* (4,177) = 1.29, *p* = 0.276, $\eta_{p}^{2}$ = 0.03] (Table 1 and Figure 1).

### Longitudinal change in NfL and GFAP

**Figure 2:**
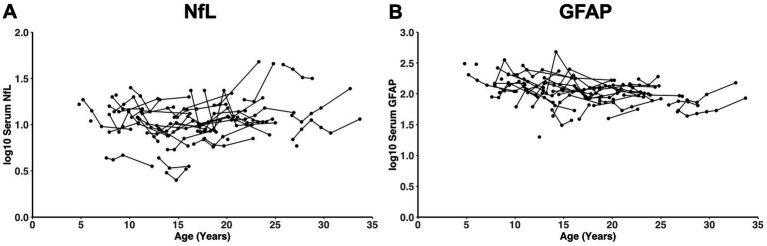
There was no significant change over time in log_10_ serum NfL **(A)** or GFAP **(B)** levels in the Wolfram group (*n* = 37) adjusting for both age at the first visit and sex (*p* = 0.279 and *p* = 0.830, respectively). Each dot is a timepoint with dots connected by lines representing an individual. NfL, neurofilament light chain; GFAP, glial fibrillary acidic protein.

There was no significant effect of time on log_10_ NfL levels in the Wolfram group using a random coefficients mixed effects model (*β* = −0.0013, SE = 0.004, *p* = 0.755) (Figure 2). The annual rate of change was not significantly different from zero after adjusting for age at the first visit (*β* = 0.0058, SE = 0.005, *p* = 0.263), nor after adjusting for both age at the first visit and sex (*β* = 0.0056, SE = 0.005, *p* = 0.279). In contrast, there was an effect of time on GFAP levels in the Wolfram group (*β* = −0.0137, SE = 0.004, *p* = 0.001) (Figure 2). However, the annual rate of change was not significantly different from zero after adjusting for age at the first visit (*β* = 0.0014, SE = 0.006, *p* = 0.812), nor after adjusting for both age at the first visit and sex (*β* = 0.0013, SE = 0.006, *p* = 0.830).

As shown in Table 2, one sample t-tests of rates of change in NfL and GFAP levels were consistent with these measures being stable over time, while all clinical measures and regional brain volumes revealed deterioration over time, as previously reported (Hoekel et al., 2018; Lugar et al., 2019; Samara et al., 2020; O’Bryhim et al., 2022), with the exception of cerebellar white matter volume.

**Table 2:** Serum NfL and GFAP levels, clinical measures, and regional brain volumes in Wolfram syndrome at time 1 and longitudinally

Measures	Time 1 n	Time 1	Longitudinal n	Average across time	Rate of change	Rate of change (*p*)	Median tps (range)
Median (IQR)							
NfL Raw (pg/mL)	45	11.5 (7.9–15.2)	37	11.1 (7.9–14.2)	0.21 ± 2.42	0.595	4 (1–6)
NfL log10	45	1.1 (0.9–1.2)	37	1.0 (0.9–1.2)	0.00 ± 0.05	0.931	4 (1–6)
GFAP Raw (pg/mL)	45	117.9 (77.4–158.3)	37	112.6 (83.9–141.4)	−3.03 ± 11.70	0.124	4 (1–6)
GFAP log10	45	2.1 (1.9–2.2)	37	2.0 (1.9–2.2)	−0.01 ± 0.04	0.225	4 (1–6)
Mean ± SD							
WURS Physical Subscale Score	41	4.46 ± 4.43	37	–	1.25 ± 2.39	**0.003**	4 (1–6)
Visual Acuity (LogMAR)	44	0.61 ± 0.47	36	–	0.08 ± 0.09	**<0.001**	3.5 (1–6)
UPSIT Total Score	45	25.16 ± 7.21	37	–	−1.03 ± 1.54	**<0.001**	4 (1–6)
Pons Volume (mm^3^)	32	7599.35 ± 917.99	26	–	−89.82 ± 95.21	**<0.001**	3 (1–6)
Brainstem Volume (mm^3^)	32	14186.16 ± 1387.89	26	–	−117.59 ± 168.00	**0.001**	3 (1–6)
Thalamus Volume (mm^3^)	32	6166.09 ± 377.89	26	–	−47.81 ± 90.17	**0.012**	3 (1–6)
Cerebellar Gray Volume (mm^3^)	32	47782.22 ± 3408.04	26	–	−271.18 ± 418.53	**0.003**	3 (1–6)

NfL, neurofilament light chain; GFAP, glial fibrillary acidic protein; IQR, interquartile range; WURS, Wolfram Unified Rating Scale; UPSIT, University of Pennsylvania Smell Identification Test; tps, time points; – indicates not applicable; Bold *p*-values indicate statistical significance at *α* = 0.05.

### Correlations between log_10_ NfL and clinical and neurodegeneration variables

**Figure 3:**
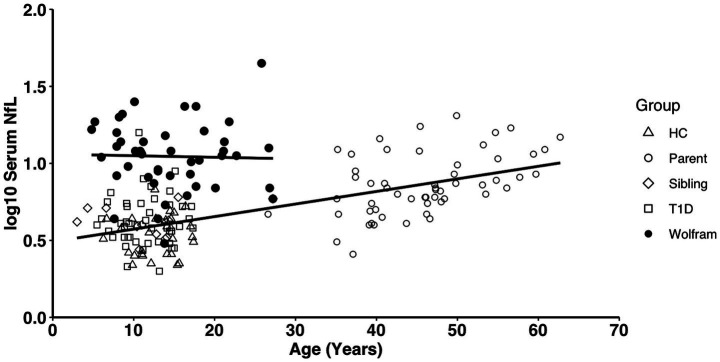
Age correlated with log_10_ serum NfL levels within the control groups (collapsed) (*p* < 0.001, *R*^2^ = 0.43) but not in the Wolfram group (*p* ≥ 0.68, *R*^2^ = 0.001). NfL, neurofilament light chain; HC, healthy control; T1D, type 1 diabetes.

Within the Wolfram group, log_10_ NfL levels were not correlated with age (*r*_45_ ≤ 0.06, *p* ≥ 0.68), whereas within the controls, age was highly correlated with log_10_ NfL levels (log_10_: *r*_142_ = 0.65, *p* < 0.001) (Figure 3).

Within the Wolfram group, higher log_10_ NfL levels at time 1 were related to lower UPSIT scores at time 1 (*r*_45_ = −0.37, *p* = 0.012); however, one participant had a value that may have overly influenced the result. While not a statistical outlier, the correlation was reduced after that participant was removed from the analysis (*r*_44_ = −0.26, *p* = 0.095). There was a marginally significant relationship between higher log_10_ NfL levels at time 1 with lower brainstem volume (*r_32_* = −0.35, *p* = 0.052), which remained marginal after controlling for age and sex (*r_28_* = −0.35, *p* = 0.061). No other significant correlations were found (*r*_26-45_ ≤ ± 0.24, *p* ≥ 0.154).

## Discussion

This study investigated the potential for serum NfL and GFAP to be used as alternative, blood-based monitoring biomarkers in Wolfram syndrome. We found that while serum NfL was consistently elevated in Wolfram syndrome compared to control groups, even after controlling for potentially confounding variables, serum GFAP was not. The NfL effect in Wolfram compared to controls was fairly large but consistent, regardless of model or number of covariates ( $\eta_{p}^{2}$ ranged from 0.27–0.52 and mean NfL levels ranged from 21 to 98% higher in Wolfram, depending on which covariates were used). Serum NfL levels did not change over time in Wolfram syndrome and did not show any reliable correlations with disease progression or neuroimaging-based measures of neurodegeneration. This pattern of findings indicates that NfL levels may be sensitive to the disease state but do not reflect individual differences in neurological presentation or trajectories.

To further strengthen this interpretation, raw NfL levels in the Wolfram group [median interquartile range (IQR) serum NfL at time 1 = 11.5 (7.9, 15.2) pg/mL] were elevated compared to previously established reference intervals from multiple studies in healthy children, adolescents, and young adults (ages 0 to 30 years) using the Simoa platform, and these median levels in healthy controls were consistent with ours [serum/plasma NfL = 3.6 to 5.1 pg/mL vs. serum NfL = 3.5 to 4.1 pg/mL, respectively] (Reinert et al., 2020; Chen et al., 2021; Nitz et al., 2021; Bornhorst et al., 2022; Abdelhak et al., 2023; Geis et al., 2023; Schjørring et al., 2023; Stukas et al., 2024; Sukhonpanich et al., 2025). Furthermore, serum NfL levels in individuals with Wolfram syndrome are similar to levels seen in other neurological disorders in children and adolescents, including untreated pediatric MS [median serum NfL = 19.0 to 21.1 pg/mL] (Reinert et al., 2020; Huppke et al., 2023; Ziaei et al., 2023) and asymptomatic spinocerebellar ataxia type 3 [median (IQR) serum NfL = 12.2 (10.2, 13.9) pg/mL] (Peng et al., 2020). NfL levels in the Wolfram group also overlap with plasma NfL levels in adults (mean age = 57.4 years) with type 1 diabetes [mean (SD) plasma NfL = 13.3 (6.7) pg/mL] (Sampedro et al., 2020).

Despite being elevated, serum NfL levels did not correlate with any of the key neurological or neurodegeneration variables known to be affected in Wolfram syndrome. This lack of findings was surprising given that our previous pilot study found several significant, albeit weak correlations (Eisenstein et al., 2022). This discrepancy could be due to several differences between these studies. For example, we have a higher number of participants in this sample, which could have washed out marginal effects from the previous analysis. In addition, we used data from different timepoints for analyses compared to the previous study. Finally, as with the marginal correlation we found between serum NfL levels and brainstem volume at time 1, it may be that our results were simply underpowered, given the small sample size of a rare disease. Nonetheless, the failure to find any predictive value of NfL levels for clinical symptoms or neurogenerative measures indicate that NfL cannot be used to substitute for these measures in clinical trials.

NfL levels remained elevated but stable over time in Wolfram syndrome, suggesting that neuroaxonal damage may be occurring at a constant, higher rate than controls rather than accelerating over time. This pattern is also seen in the steady, linear reductions in regional brain volumes in this group (Lugar et al., 2019). In addition, this pattern is similar to our previous pilot study on Wolfram syndrome over a shorter time period (1.8 months, Eisenstein et al., 2022), to patterns in spinocerebellar ataxia over a two-year period (Coarelli et al., 2021) and to patterns in cognitively impaired older adults over a 4-year time period (Benedet et al., 2020).

Concurrent elevations in NfL and GFAP levels are commonly seen in individuals with aging-related neurodegenerative disorders relative to controls (Zeitlberger et al., 2018; Watanabe et al., 2019; Schindler et al., 2021; Sanchez et al., 2024; Saucier et al., 2024; Wang et al., 2024). Thus, it was unexpected to find that GFAP levels were not elevated in our Wolfram group compared to controls. Interestingly, a recent study in Niemann-Pick Type C (NPC) disease, a rare genetic neurodegenerative lysosomal storage disorder (Eratne et al., 2025) found a similar discrepancy. NPC, like Wolfram syndrome, is caused by a genetic mutation with heterogeneous clinical presentations during neurodevelopment that can include cerebellar and thalamic atrophy (Masingue et al., 2017; Las Heras et al., 2023). This discrepancy between the behavior of these serum markers in neurodegeneration during neurodevelopment vs. aging suggests that underlying neurodevelopmental processes may somehow suppress or interfere with reactive astrogliosis as measured by GFAP.

Neurofilament light chain levels display a U-shaped curve over the lifespan in healthy individuals with high levels in infants that decrease throughout childhood and level off into young adulthood followed by a steady increase with age (Reinert et al., 2020; Nitz et al., 2021; Beerepoot et al., 2022; Abdelhak et al., 2023; Cooper et al., 2023; Geis et al., 2023; Schjørring et al., 2023; Bavato et al., 2024; Rodero-Romero et al., 2024; Stukas et al., 2024). In normal aging, higher NfL levels, both cross-sectionally and longitudinally, correlate with brain atrophy, likely reflecting typical neuronal loss with advancing age (Mielke et al., 2019; Khalil et al., 2020; Karoly et al., 2021; Marks et al., 2021; Rübsamen et al., 2021; Beydoun et al., 2023). The unaffected sibling control group in our study had serum NfL levels that were roughly half of the levels seen in the parent controls. In addition, the similarly aged unrelated healthy control group and newly diagnosed T1D control group had comparable NfL levels with the sibling control group, which were all in line with established reference NfL levels for young healthy individuals (Reinert et al., 2020; Nitz et al., 2021; Abdelhak et al., 2023; Geis et al., 2023; Schjørring et al., 2023; Stukas et al., 2024). The parent control group in our study also had NfL levels consistent with the established adult reference range for the Quanterix/Simoa platform (Harp et al., 2019) and other studies with similarly aged adult controls (Korley et al., 2019; Harp et al., 2022), and older age correlated with higher NfL levels.

The major strength of this study is the rich, longitudinal phenotyping and quantitative neuroimaging measures of neurodegeneration in Wolfram syndrome, an ultra-rare disorder. Serum biomarker levels in Wolfram syndrome were compared to multiple control groups, including unaffected siblings and parents of the Wolfram participants and unrelated healthy controls and youth with T1D, and all samples were assayed at the same time by a single lab. However, there are several limitations to the current study. While this is the largest study to date of blood biomarkers in Wolfram syndrome, the study sample is relatively small compared to more common neurodegenerative diseases. In addition, the T1D control group was newly diagnosed and thus had a much shorter average diabetes duration (~6 months) compared to the Wolfram group (~9 years). Future studies may want to obtain a T1D group matched to the Wolfram syndrome group on age, sex, glucose control and diabetes duration to avoid this issue.

## Conclusion

In summary, this study has demonstrated that serum NfL but not GFAP levels are elevated in individuals with Wolfram syndrome independent of family membership, age, diabetes status, and kidney function. Although elevated, NfL levels do not correlate reliably with any neurological or neurodegeneration measures, indicating that serum NfL may have limited utility as a monitoring biomarker. The lack of change in NfL levels over time in Wolfram syndrome may indicate that neurodegeneration is occurring at a constant rate rather than accelerating over time. Overall, this study shows that serum NfL may be a reflection of ongoing neurodegeneration in Wolfram syndrome but may not be sensitive or specific enough to track disease progression. However, it could still be informative in future clinical trials as a treatment-response marker.

## Data Availability

The raw data supporting the conclusions of this article will be made available by the authors, without undue reservation.
